# Development of a real-time quantitative PCR assay for detection of a stable genomic region of BK virus

**DOI:** 10.1186/1743-422X-7-295

**Published:** 2010-10-29

**Authors:** Kosuke K Iwaki, Suhail H Qazi, Jean Garcia-Gomez, Deanna Zeng, Yasuhiro Matsuda, Kazuko Matsuda, Monica E Martinez, Mieko Toyoda, Arputharaj Kore, Wesley T Stevens, Miroslaw Smogorzewski, Daisuke D Iwaki, Yasir Qazi, Yuichi Iwaki

**Affiliations:** 1 Metic Transplantation Laboratory, USC, Keck School of Medicine, Los Angeles, CA, USA; 2 Transplant Immunology Laboratory, Cedars-Sinai Medical Center, Los Angeles, CA, USA; 3 Division of Transplantation, Department of Surgery, Loma Linda University Medical Center, Loma Linda, CA, US; 4 Department of Pathology, Loma Linda University Medical Center, Loma Linda, CA, US; 5 Internal Medicine, USC, Keck School of Medicine, Los Angeles, CA, US

## Abstract

**Background:**

BK virus infections can have clinically significant consequences in immunocompromised individuals. Detection and monitoring of active BK virus infections in certain situations is recommended and therefore PCR assays for detection of BK virus have been developed. The performance of current BK PCR detection assays is limited by the existence of viral polymorphisms, unknown at the time of assay development, resulting in inconsistent detection of BK virus. The objective of this study was to identify a stable region of the BK viral genome for detection by PCR that would be minimally affected by polymorphisms as more sequence data for BK virus becomes available.

**Results:**

Employing a combination of techniques, including amino acid and DNA sequence alignment and interspecies analysis, a conserved, stable PCR target region of the BK viral genomic region was identified within the VP2 gene. A real-time quantitative PCR assay was then developed that is specific for BK virus, has an analytical sensitivity of 15 copies/reaction (450 copies/ml) and is highly reproducible (CV ≤ 5.0%).

**Conclusion:**

Identifying stable PCR target regions when limited DNA sequence data is available may be possible by combining multiple analysis techniques to elucidate potential functional constraints on genomic regions. Applying this approach to the development of a real-time quantitative PCR assay for BK virus resulted in an accurate method with potential clinical applications and advantages over existing BK assays.

## Background

BK virus (BKV), along with JC virus (JCV) and Simian virus 40 (SV40), are members of the family *Polyomaviridae*. BKV and JCV are ubiquitous in human populations worldwide with a seroprevalence in adults of 70%-80% [1-4]. They establish persistent, latent infections and are capable of reactivating in immunosuppressed hosts [5-7]. BKV in particular is recognized as a significant cause of allograft failure in renal transplant recipients [8]. In addition, these viruses may also be associated with renal dysfunction in nonrenal transplant recipients [9,10]. Prospective monitoring of patients at risk for BKV-associated nephropathy (BKVAN) or BKV associated morbidity may identify those patients with active infection before renal function deteriorates [11-13]. Early identification of active BK infection in transplant recipients is advantageous for controlling BKV replication and preventing BKVAN via reduction of immunosuppression or use of cidofovir antiviral therapy [14,15].

BKV screening protocols and quantitative BKV testing are increasingly performed in molecular virology laboratories. Guidelines for quantitative cutoffs for nucleic acid tests for BK viruria and BK viremia that indicate the need for additional clinical testing were proposed in 2005 [5]. However, the usefulness of these cutoffs is hampered by the lack of standardized assays, uniform external viral standard, the existence of viral subtypes and the presence of viral polymorphisms. Primers and probes developed for quantitative BKV testing based on limited available sequence data from few viral isolates suffer reduced performance in detection of viral isolates with sequence variations in these regions. This in turn can lead to inconsistent detection of virus, inaccurate quantitation of viral load and difficulty comparing results between assays.

We describe an approach for the development of a stable nucleic acid assay when limited nucleotide sequence information is available. We report that interspecies amino acid and nucleotide sequence analysis, in conjunction with intraspecies nucleotide sequence alignment, can elucidate genomic regions that may be under potential functional constraints and that these regions can be targeted for primer and probe design to improve assay performance.

## Results

### Intraspecies nucleotide sequence variation analysis of BK viral genes

**Figure 1:**
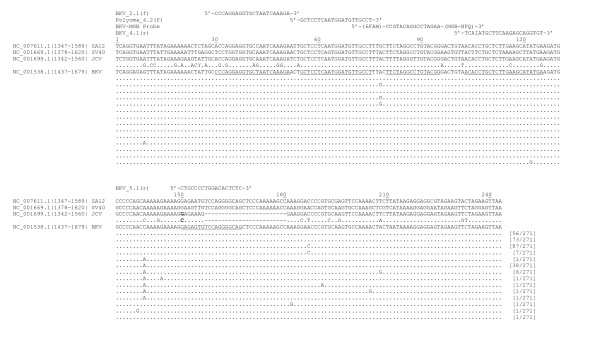
**Alignment of the nucleotide sequence corresponding to the VP2(VP3) C-terminus region**. Nucleotide alignment of reference sequences for Simian Agent 12 (NC_007611.1; nucleotides 1347-1589), Simian virus 40 (NC_001669.1; nucleotides 1378-1620), JCV (NC_001699.1; nucleotides 1342-1560), and BKV (NC_001538.1; nucleotides 1437-1679) is shown. The positions of nucleotide polymorphisms among 487 JCV isolates relative to the reference sequence are shown below the JCV reference sequence. The guanine at position 150 (bold) is replaced by cytosine in six out of 487 JCV isolates. The locations of polymorphisms among 271 BKV isolates are shown below the BKV reference sequence and the number of BKV isolates with that particular set of polymorphism(s) is given in brackets. The positions of the primers (underlined) and probe (boxed) are indicated on the BKV reference sequence. There are no observed polymorphisms at the Polyoma_4.2(f) forward primer in either BKV or JCV sequences available at this time. There are no known polymorphisms at the relative position of the BKV_MGB probe (boxed) among either the BKV isolates or JCV isolates. There are three nucleotide differences between JCV and BKV at the probe binding site. There are three nucleotide differences between SV40 and BKV at the BKV_5.1(r) reverse primer binding site.

The initial step in identifying a stable region of the BK viral genome for assay development was nucleotide sequence variation analysis of the BK virus (BKV) genes, performed on 157 to 160 BKV isolates using ClustalW2 [16] and MUSCLE [17] analyses. The total number of polymorphisms per gene based on multiple nucleotide sequence alignments for the six BKV genes is given in Table 1. These analyses show that sequence variants are widespread throughout the BKV genome and occur in all 6 viral genes. Of note, in comparison to most other areas of the BKV genome, there were substantially fewer sequence variants corresponding to the C-terminus of the VP2 gene. The distribution of sequence variants in the C-terminus region of the VP2 is presented in Figure 1.

**Table 1:** Polymorphisms in BKV Genes

	# of SNPs*	Gene size (bp)
Agno protein	48	201
Small - T antigen	53	519
Large - T antigen	316	2088
VP - 1	179	1089
VP - 2	106	1056
VP - 3	86	699

*ClustalW2 alignment of available nucleotide sequences in GENBANK. The size of each respective gene is based on the NCBI reference sequence (accession number: NC_001538).

Several regions of the BKV genome demonstrated either 100% homology or limited to minor mismatches among the BKV isolates being compared. These regions, including sections of the C-terminal helicase domain of the Large-T antigen, sections of the N-terminal heat shock protein DNA-J of the Large-T antigen, sections of the N-terminus of the VP2 gene, and sections of the C-terminus of the VP2/VP3 gene, were considered equally suitable targets for assay development at this stage.

### Interspecies amino acid analysis of potential assay target regions in *Polyomaviridae *family members

**Figure 2:**
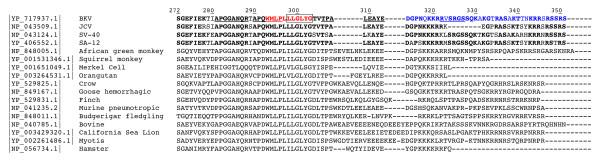
**Amino Acid Sequence Alignment of the VP2 and VP3 C-terminus from representative viruses of the *Polyomaviridae *Family**. Alignment of the amino acid sequence of the VP2/VP3 C-terminus was constructed using ClustalW2 from reference sequences of 17 different members of the *Polyomaviridae *family. BKV, JCV, SV40 and SA12 sequences were compared and residues that are conserved between at least three out of these four closely related members of the *Polyomaviridae *family are indicated in bold. The alpha-helix region of the BKV VP2/VP3 gene is shown in red. The location of the primers (underlined) and the probe (boxed) are indicated. The region in the BKV VP2/VP3 gene having two reading frames due to the overlap with the N-terminus of the VP1 gene is indicated in blue. The NLS and DNA binding region are located downstream of the alpha helix sequence. Residues are numbered according to the reference sequence for the BKV VP2 gene (YP_717937.1).

To further evaluate the potential assay target regions identified by intraspecies nucleotide analysis and to develop supporting evidence for the sequence stability of these regions, an interspecies amino acid sequence comparison of each of the regions was performed. Among the four potential target regions, the VP2 C-terminus region was distinguished from the others due to the presence of a number of motifs and recognized structural elements including: (1) an alpha helix at the C-terminus [18], (2) a known VP1 interaction region in the C-terminus [18], (3) a series of basic amino acid residues comprising a nuclear localization signal (NLS) [19-22], (4) a DNA binding region [23,24] and (5) a shared open reading frame due to the overlap with the N-terminus of VP1 [25,26]. Examination of members of the *Polyomaviridae *family revealed that the VP2 C-terminus region and the alpha-helical region in particular, are conserved across multiple members of the *Polyomaviridae *family, suggestive of a region under a high degree of structural and functional constraint (Figure 2). It also confirmed previous observations [18] that the residues downstream of the alpha-helix region exhibit more variability across members of the *Polyomaviridae *family. Lastly, members of the *Polyomaviridae *family that are more closely related to BKV (i.e. JCV, Simian Agent [SA]12, and SV40) exhibit stretches of amino acids downstream of the alpha-helical region that are also conserved (Figure 2). The degree of sequence conservation in the VP2 C-terminus region and the presence of known structural and functional elements suggest a reduced likelihood of sequence variation. In addition, a section of the VP2 C-terminus, (residues 324-330, SRGSSQK), is conserved in BKV, SV40, and SA12, but absent in JCV. Taken together, these results support the C-terminus of VP2 as a sequence stable target for assay development that could also be specific for BKV, relative to JCV.

### Interspecies and intraspecies comparison of nucleotide sequences of the assay target region

The regions of the VP2 C-terminus identified by amino acid alignment were further analyzed by intraspecies sequence analysis of JCV isolates and interspecies nucleotide sequence alignment among very closely related viruses (Figure 1). This enabled cross species comparison of the relative locations of polymorphisms within the selected target region. Focus was primarily directed at comparing the BKV and JCV sequences as they are clinically important viruses that are taxonomically closely related and there were a considerably greater number of sequences available for JCV.

Intraspecies nucleotide sequence analysis of the VP2 gene identified several stretches of conserved codons among BKV isolates (Figure 1). The nucleotide sequence 1 to 135 (residues 272-316, Figure 2) is conserved among BKV sequences, containing only three polymorphic positions. These polymorphisms represent synonymous mutations at Glu (residue 274) and Leu (residue 297) and a non-synonymous mutation at residue 312 (Tyr to Cys). Two other stretches of conserved nucleotides were observed: the stretch of 36 nucleotides 145 to 180 corresponding to KRRVSRGSSQKA (residues 320-331, Figure 2) and the stretch of 33 nucleotides 211 to 243 corresponding to NKRRSRSSR (residues 342-351, Figure 2) and stop codon. Polymorphisms were not observed among available BKV sequences for these latter two regions.

Interspecies nucleotide comparison of known polymorphic sites in VP2 between BKV and JCV revealed a stretch of 41 nucleotides 55 to 95 that is devoid of polymorphisms aside for a single polymorphism for BKV at nucleotide 78. This stretch of nucleotides corresponds to the alpha-helix region and the three adjacent up-stream residues APQWMLPLLLGLYG (residues 290-303, Figure 2). With the exception of a single nucleotide polymorphism at position 9, the sequence upstream of nucleotide position 55 is conserved among BKV sequences. However, a significant number of polymorphic sites are observed among the corresponding JCV nucleotide sequences.

Based on these results, primers and probe were designed targeting the nucleotide sequence corresponding to the VP2 C-terminus region (Figure 1).

### Design of PCR assay primers and probe

The assay forward primer (Polyoma_4.2 [sense strand]) was designed to correspond to nucleotides 55 to 75 of BKV sequence (Figure 1), which contains the codons for part of the alpha-helix region. No polymorphisms were observed within BKV or JCV or between BKV and JCV sequences. An alternate forward primer (BKV_2.1 [sense strand]) was designed corresponding to nucleotides 30 to 51 of BKV sequence (Figure 1). This sequence reflects a region of interspecies amino acid conservation among BKV, JCV, SV40 and SA12 reference strains (residues 281 to 288, Figure 2). Although the nucleotide analyses indicate it is conserved among the available BKV isolates, this stretch of nucleotides display multiple polymorphisms among the available JCV sequences.

The assay reverse primer (BKV_5.1 [anti-sense strand]) was designed to correspond to nucleotide sequences 150 to 167 of the BKV sequence (Figure 1). This sequence contains the codons for amino acid residues SRGS (residues 324-327, Figure 2) that are deleted in JCV. No polymorphisms are observed along this stretch of nucleotides among the available BKV sequences. Three consecutive nucleotide differences exist between the BKV and SV40 reference sequences within the primer (Figure 1). An alternate reverse primer (BKV_4.1 [anti-sense strand]) was designed corresponding to nucleotides 103 to 125 of the BKV sequence (Figure 1). There is a single polymorphism within BKV isolates in the primer sequence, resulting in use of the universal deoxyinosine nucleotide at the corresponding position in the primer. Several polymorphisms were observed between BKV and JCV reference sequences corresponding to this primer.

The probe sequence (BKV-MGB Probe [anti-sense strand]) corresponds to nucleotide sequences 80 to 95 (Figure 1). This sequence contains the codons for the alpha-helix residues LLGLYG, conserved in the SA12, SV40, JCV and BKV (Figure 2). There are a total of three nucleotide differences between BKV and JCV reference sequences at the probe binding site (Figure 1) and this difference is present in all BKV and JCV isolates. No polymorphisms in the probe sequence were observed between BKV and SV40 reference sequences.

The nucleotide comparisons described above allowed design of a combination of primers and probes that targets a sequence stable region yet provides specificity for BKV relative to JCV and SV40.

### Development and validation of a real-time quantitative PCR assay for the VP2 region of BK virus

**Figure 3:**
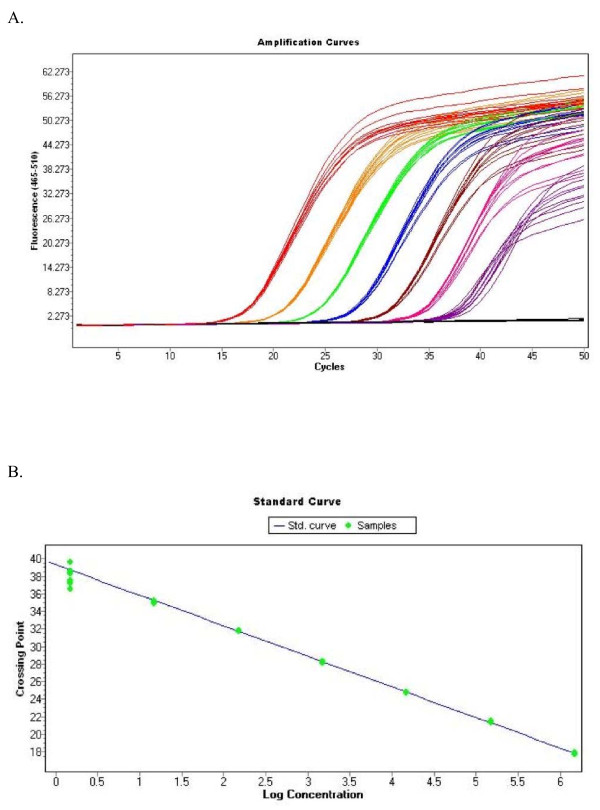
**BKV Real-Time Quantitative PCR**. The BKV standard was serially diluted prior to being assayed by real-time quantitative PCR. A. Raw amplification curves from precision study with primers BKV_5.1(r) and BKV_4.2(f). Input BKV DNA was 10 μl at concentrations of 10^6 ^copies/μl, 10^5 ^copies/μl, 10^4 ^copies/μl, 10^3 ^copies/μl, 10^2 ^copies/μl, 10 copies/μl and <10 copies/μl. B. Standard curve. Results of the regression were: slope -3.484, y-intercept 39.05 and efficiency 1.936. Similar results were obtained with other primer combinations.

Once a stable region of the BKV genome was identified, a real-time quantitative PCR assay was developed. Serial dilution experiments were undertaken to evaluate assay performance. The BKV assay was linear over a 6-log range from 10^7 ^copies/reaction to 10^1 ^copies/reaction (Figure 3).

The sensitivity of the BKV assay was evaluated by testing a BKV standard (Advanced Biotechnologies Inc., MD) at known concentrations of 1.5, 15, 75 and 150 copies/reaction. Analytical sensitivity was determined by the lowest serial dilution consistently detectable in replicate reactions. The assay detected BKV with 100% (30/30) sensitivity at BKV copy levels of 15 copies/reaction and greater (Table 2). BKV at a copy level of 1.5 copies/reaction was detected in 2 to 3 of 10 replicates (20-30%). Therefore the analytical sensitivity of the assay is 15 copies/reaction, which is equivalent to 450 copies/ml given a 200 μl extraction volume and an elution volume of 60 μl. The lower limit of detection of the assay, which is detectable but not reliably quantifiable, is 1.5 copies/reaction.

**Table 2:** Sensitivity of the BKV Real-Time Quantitative PCR Assay

		Crossing Point								
		
		BKV_5.1/Polyoma_4.2			BKV_4.1/Polyoma_4.2			BKV_4.1/BKV_2.1		
		
Copies per Reaction*	# of Reactions	Mean	SD	CV	Mean	SD	CV	Mean	SD	CV
150	10	36.16	0.75	2.06	36.46	0.33	0.90	37.19	0.31	0.82
75	10	37.75	0.94	2.48	37.55	0.51	1.35	38.52	0.38	0.98
15	10	39.86	1.05	2.63	39.22	0.84	2.15	40.51	1.31	3.24
1.5**	10	40.84	0.58	1.42	41.08	0.22	0.53	43.75	2.17	4.96

SD = standard deviation; CV is a percentage

*5 replicates of negative control were all negative

* *only 2 to 3 of 10 replicates for each primer combination had crossing points above background

The specificity of the BKV assay was evaluated by testing replicates of JCV DNA standard (Advanced Biotechnologies) at concentrations of 1.4 × 10^4 ^copies/reaction and mixed samples containing BKV at various concentrations combined with JCV at 7 × 10^3 ^copies/reaction compared to the same concentrations of BKV without any JCV present. No false positive results (0/10) were observed when testing JCV samples (Table 3). In addition, no inhibition of BKV detection in the presence of JCV was observed, as crossing point (Cp) values for BKV in mixed samples with JCV were similar for the same concentrations of BKV alone.

**Table 3:** Specificity of the BKV Real-Time Quantitative PCR Assay

			Crossing Point		
			
			BKV_5.1/Polyoma_4.2		
			
Copies BKV/Reaction*	Copies JCV/Reaction*	# of Reactions	Mean	Std Dev	CV (%)
1.5 × 10^7^	7.0 × 10^3^	10	18.43	0.07	0.37
1.5 × 10^6^	7.0 × 10^3^	10	21.99	0.02	0.11
1.5 × 10^5^	7.0 × 10^3^	10	25.61	0.03	0.10
1.5 × 10^4^	7.0 × 10^3^	10	29.14	0.04	0.15
1.5 × 10^3^	7.0 × 10^3^	10	33.06	0.08	0.25
1.5 × 10^2^	7.0 × 10^3^	10	36.74	0.66	1.80
1.5 × 10^1^	7.0 × 10^3^	10	39.61	1.22	3.08
1.5 × 10^7^	0	2	18.41	0.02	0.12
1.5 × 10^6^	0	2	22.01	0.08	0.35
1.5 × 10^5^	0	2	25.69	0.01	0.06
1.5 × 10^4^	0	2	29.28	0.08	0.27
1.5 × 10^3^	0	2	33.03	0.05	0.15
1.5 × 10^2^	0	2	36.91	0.27	0.73
1.5 × 10^1^	0	2	39.94	1.97	4.94
0	1.4 × 10^4^	10	---	---	---
Negative Control		5	---	---	---

The precision of the assay was evaluated based upon the reproducibility of the Cp values for a BKV DNA standard, which was serially diluted to concentrations of 1.5 × 10^6 ^copies/μl, 1.5 × 10^5 ^copies/μl, 1.5 × 10^4 ^copies/μl, 1.5 × 10^3 ^copies/μl, 1.5 × 10^2 ^copies/μl, 1.5 × 10^1 ^copies/μl and 1.5 × 10^0 ^copies/μl. A total of ten replicates for all concentrations were tested. The coefficient of variation (CV) in the Cp for all concentrations tested was ≤ 2.3% (Table 4), verifying a high degree of assay precision across the entire range of concentrations tested.

**Table 4:** Precision of the BKV Real-Time Quantitative PCR Assay

		Crossing Point								
		
		BKV_5.1/Polyoma_4.2			BKV_4.1/Polyoma_4.2			BKV_4.1/BKV_2.1		
		
Copies/Reaction*	# of Reactions	Mean	CV (%)	Std Dev	Mean	CV (%)	Std Dev	Mean	CV (%)	Std Dev
1.5 × 10^7^	10	17.77	0.30	0.05	17.68	0.89	0.16	15.89	1.33	0.21
1.5 × 10^6^	10	21.35	0.31	0.07	21.12	0.59	0.13	19.45	0.50	0.10
1.5 × 10^5^	10	24.77	0.15	0.04	24.56	0.71	0.17	22.82	1.02	0.23
1.5 × 10^4^	10	28.25	0.21	0.06	28.09	0.43	0.12	26.27	0.72	0.19
1.5 × 10^3^	10	31.77	0.20	0.06	31.62	0.67	0.21	29.76	0.57	0.17
1.5 × 10^2^	10	35.03	0.37	0.13	35.09	0.53	0.19	33.55	0.94	0.32
1.5 × 10^1^	10	38.03	2.30	0.88	38.41	1.50	0.58	37.33	1.79	0.67

*5 replicates of negative control were all negative

## Discussion

There have been previous efforts to develop real-time PCR based BKV quantification assays [27,28]. However, many of these assays were developed when the number of available DNA sequences in public databases was quite limited. Although multiple sequence alignments are routinely used to identify stable target regions for assay development, sequence variants have been observed in these regions as more strains and sequences become available. Therefore, when the number of isolates and sequences is limited, merely selecting a target region for assay development based on intraspecies nucleotide sequence alignments alone may not be sufficiently predictive of interstrain sequence conservation to result in a sensitive and specific assay.

We proposed that identifying areas of the genome that are conserved within and between species could be used to supplement limited nucleotide sequence information when designing assays. Target regions identified via standard intraspecies sequence alignment of full genome sequences from available isolates were further evaluated by interspecies amino acid analysis to select a single target region. The interspecies amino acid alignments assisted in narrowing down potential target regions by identifying areas containing functionally and/or structurally constrained sequences. Conservation in amino acid sequence among closely related viruses does not necessarily preclude the possibility of non-synonymous substitutions. Functional and structural constraints, however, do suggest that there would likely be fewer number of amino acid residues that can reside at that particular position than if the same region was under less stringent functional or structural constraint.

However, mutations in the nucleotide sequences giving rise to synonymous substitutions can potentially have equally adverse effects on the performance of a PCR assay. Possible mechanisms for constraints limiting synonymous mutations may include overlapping reading frames, especially if those overlapping regions are functionally or structurally important. Identifying potential cold spots in the genome that are less likely to undergo synonymous substitutions is a significant challenge and a major obstacle to developing a robust PCR-based viral assay. Intra-species nucleotide alignment of available BKV sequences was followed by detailed intra- and interspecies nucleotide analysis of the target region to aid in designing assay primers and probe.

In the present work, the utilization of the intraspecies nucleotide alignment of BKV with the intraspecies nucleotide alignment of a closely related virus, JCV, for which a significant number of sequences were available, proved to be useful in substantiating that the Polyoma_4.2(f) and the BKV-MGB probe binding regions within BKV was less likely to experience nucleotide sequence variation. The lack of polymorphisms at the corresponding positions in both BKV and JCV isolates provides stronger evidence for sequence conservation than does intra-species nucleotide analysis of BKV isolates alone. Conversely, the forward primer BKV_2.1(f) is reserved for use only as a secondary backup to Polyoma_4.2(f) due to the extent of polymorphisms seen among JCV isolates along the nucleotide stretch corresponding to the BKV_2.1(f) binding site.

Since the assay was first developed using ~160 BKV sequences, the number of available BKV complete genome sequences increased to 271 (Additional file 1). As presented in the results section, the increase in BKV sequence data did not demonstrate increased sequence variation in the region targeted by this analysis, validating the approach of incorporating the additional analyses discussed herein when designing a real-time PCR assay.

The amino acid and nucleotide sequence analyses that were performed identified the VP2 C-terminus as a probable functionally conserved region, consistent with the presence of an alpha-helix [18] and nuclear localization signal [19-22]. Targeting the VP2 gene for assay development is in contrast to the location of primers/probes for most existing BKV assays [27]. Therefore this approach was successful in identifying a region different from those targeted by other assays that have become limited with the recognition of additional sequence variations.

Using functionally constrained regions for assay development might be expected to result in lack of specificity. However, sufficient nucleotide differences existed within the stable region to design an assay specific for BKV. The three nucleotide difference between the BKV and JCV at the probe binding site was sufficient to meet assay specificity requirements by utilizing a Taqman^®^-MGB probe. By having the probe bind to the anti-sense strand, the respective positions of the inter-species nucleotide differences between the BKV and JCV were such that the minor groove binder of the Taqman^®^-MGB probe could be ideally positioned to provide a high degree of specificity. This configuration also provided the added benefit of having the probe bind to the same strand (anti-sense) as the BK virus specific reverse primer rather than on the sense strand that binds the Polyoma_4.2(f) primer, which amplifies both JCV and BKV. It should be noted that the primer combination BKV_5.1(r) and Polyoma_4.2(f) is the only primer combination that has been designed and tested to perform under a BKV and JCV co-infection scenario.

Preliminary assay performance results indicate the BKV assay has a 6-log dynamic range, a lower detection limit of 1.5 × 10^1 ^copies/reaction and an intra-assay CV of ≤ 5.0%. Good precision is important for distinguishing between assay variation and assay results that are associated with clinical treatment or indicative of the need for treatment. A wide dynamic range is significant for clinical application of the assay since the clinical need is for identification of patients with high viral load who may be at immediate risk for developing BKVAN and also for identification of low viral loads in patients being monitored for BKV reactivation. The assay also does not detect JCV and the presence of JCV does not inhibit detection of BKV at the concentrations tested, suggesting that accurate detection and quantitative results for BKV will be obtained for BKV clinical samples that are co-infected with JCV.

The relative lack of sequence variants in the VP2 gene suggests that an assay targeting this region will permit accurate detection of BKV and estimates of viral load regardless of subtype (Additional file 2). Such information is important in identifying active BKV infections in transplant recipients in time to initiate appropriate treatment. Further testing of this BKV assay is needed to fully characterize assay performance characteristics, clinical performance and to evaluate the impact of sequence variations.

## Conclusion

Identifying stable PCR target regions when limited DNA sequence data is available is possible by combining multiple analysis techniques to elucidate genomic regions under functional and structural constraints. Applying this approach to the development of a real-time quantitative PCR assay for BKV resulted in an accurate method with potential clinical applications and advantages over existing BK assays.

## Methods

### Sequence alignments: Amino Acid & DNA

A total of 185 amino acid sequences for the BKV VP2 gene were obtained from the NCBI database. The amino acid sequences of the VP2 genes for the other members of the family *Polyomaviridae *were collected for species in which the information was available. Multiple sequence alignment of the amino acid sequence of the VP2 gene was performed using ClustalW2 http://www.ebi.ac.uk.

At the time of preliminary assay development, a total of 160 nucleotide sequences of the Agno protein, 159 nucleotide sequences of the large-T antigen, 157 nucleotide sequences of the small-T antigen, and 159 nucleotide sequences of the three structural genes, VP1, VP2, and VP3 were obtained from the NCBI database http://www.ncbi.nlm.nih.gov. Multiple alignments for the sequences of the six respective genes were performed using ClustalW2 http://www.ebi.ac.uk and MUSCLE http://www.ebi.ac.uk.

The entire amplicon sequence was input into the BLAST software http://www.ncbi.nlm.nih.gov to conduct species-specific searches and to analyze intra-species polymorphisms as well as interspecies sequence variation amongst closely related, clinically significant viruses. The BLAST search and results formatting were adjusted according to the number of available sequences within the database for each specific species. The results were formatted with inquiry sequence anchored with stars for identity to generate a multiple sequence alignment of the BLAST results.

### Oligonucleotides

The theoretical Tm calculations as well as secondary structure and primer-dimer analysis for oligonucleotides were performed using OligoCalc [29] & Primer3 [30]. The Tm under the actual reaction conditions was confirmed empirically. The need for a probe was confirmed by empirically testing for primer-dimer formation using the LightCycler^®^480 SYBR Green I Master reagent as well as post-amplification gel-electrophoresis.

Primers were purchased from Invitrogen Inc. (Carlsbad, CA). Two forward and two reverse primers were designed to amplify the C-terminus region of the VP2/3 gene. The BKV_5.1(r) comprised the primary reverse primer of the assay while the BKV_4.1(r) was utilized as an alternate. The reverse primer denoted as BKV_5.1(r) comprises the nucleotide sequence 5'-CTG CCC CTG GAC ACT CTC-3'. The reverse primer denoted as BKV_4.1(r) has the sequence 5'-TCA (I)AT GCT TCA AGA GCA GGT GT-3', wherein the nucleotide (I) represents deoxyinosine. The nucleotide sequence of the two forward primers denoted as Polyoma_4.2(f) and BKV_2.1(f) are 5'-GCT CCT CAA TGG ATG TTG CCT-3' and 5'-CCC AGG AGG TGC TAA TCA AAG A-3', respectively. The Polyoma_4.2(f) comprised the primary forward primer of the assay while the BKV_2.1(f) was utilized as an alternate.

TaqMan^®^-MGB probes were supplied by Applied Biosystems Inc. (Foster City, CA). The BKV probe denoted as BKV-MGB probe (5'-6FAM-CCG TAC AGG CCT AGA A-MGB-NFQ-3') was tagged with 6FAM™ at the 5'-end and tagged with a MGBNFQ (Minor Groove Binder/Non-fluorescent quencher) at the 3'-end. The JCV probe denoted as JCV-MGB probe (5'-VIC-CGT ACA ACC CTA AAA G-MGB-NFQ-3') was tagged with VIC^® ^at the 5'-end and MGBNFQ (Minor Groove Binder/Non-fluorescent quencher) at the 3'-end.

### Viral DNA Standards

The BKV (strain MM) and JCV (strain MAD1) DNA standards were obtained from Advanced Biotechnologies Inc. (Columbia, MD), supplied at 1-2 × 10^5 ^DNA copies/μl. Viral standard at 1.5 × 10^6 ^DNA copies/μl were produced from clinical BKV isolates using TOPO^® ^TA Cloning kit (Invitrogen Inc., CA, U.S.A.). Cloned viral DNA was quantified on the Roche LightCycler^®^480 using the commercially purchased standard.

### Quantitative real-time PCR

Quantitative real-time PCR was performed on a Roche LightCycler^®^480 instrument. PCR amplification was run in a final volume of 40 μl per reaction comprising 30 μl of PCR master mix and 10 μl of sample, standard or PCR-grade water. PCR master mix for one reaction comprised of: 0.42 μM forward primer, 0.42 μM of reverse primer, 0.33 μM of TaqMan^®^-MGB probe, and 20 μl of LightCycler^® ^480 Probes Master (2×).

Thermal cycling conditions when using the reverse primer BKV_4.1 were as follows: pre-incubation at 95°C for 10 minutes; 45 cycles of a three-step PCR wherein one cycle comprising of 95°C for 10 seconds (ramp rate 4.4°C/s), 60°C for 15 seconds (ramp rate 2.2°C/s) and 72°C for 1 second (ramp rate 4.4°C/s); and ending with a cool down step at 40°C for 30 seconds. Fluorescence was measured at each cycle following the third step at 72°C.

Thermal cycling conditions when using the reverse primer BKV_5.1 were as follows: pre-incubation at 95°C for 10 minutes; 45 cycles of a three-step PCR wherein one cycle comprised 95°C for 10 seconds (ramp rate 4.4°C/s), 58°C for 20 seconds (ramp rate 2.0°C/s) and 65°C for 1 second (ramp rate 4.4°C/s); and ending with a cool down step at 40°C for 30 seconds. Fluorescence was measured at each cycle following the third step at 65°C.

BKV external standards at seven different concentrations (10^6 ^copies/μl, 10^5 ^copies/μl, 10^4 ^copies/μl, 10^3 ^copies/μl, 10^2 ^copies/μl, 10 copies/μl and <10 copies/μl) were included with each assay in order to generate the standard curve. Linear regression of the external standards and quantification of unknowns were performed using the LightCycler^® ^480 software.

### Performance characterization of assay

Specificity testing. Specificity data were generated by testing analytical samples containing BKV, JCV and mixed samples containing both BKV and JCV. Serial dilutions of BKV to result in from 1.5 × 10^1 ^copies/reaction to 1.5 × 10^7 ^copies/reaction were tested individually (2 replicates of each concentration) and in the presence of 7 × 10^3 ^copies/reaction of JCV (10 replicates of each concentration). JCV at 1.4 × 10^4 ^copies/reaction (10 replicates) was also tested individually. A total of 94 specificity samples were tested. Five replicates of a negative control were also tested.

Analytical sensitivity evaluation. Analytical sensitivity samples comprised of BKV at concentrations of 150, 75, 15 and 1.5 copies/reaction were used to evaluate assay sensitivity. The BKV samples were prepared by serial dilution of a BKV standard. A total of 120 samples, 10 replicates of each concentration with 3 primer combinations, were tested in the assay. Five replicates of a negative control were also tested.

Assay Precision. The intra-assay precision of the BKV assay was evaluated. The samples were constructed by serial dilution of BKV standard and contained from 1.5 × 10^1 ^copies/reaction to 1.5 × 10^7 ^copies/reaction. Multiple replicates (10) of the seven different concentrations of the BKV standard were tested with 3 primer combinations in one run and the variance in the Cp (crossing point) calculated to determine the reproducibility of the assay. A total of 210 samples were tested. Five replicates of a negative control were also tested.

## Competing interests

The primary author is listed as an inventor in a published International Patent Application filed by Metic Immunogenetic Consultant, Inc., entitled "Detection of Polyomavirus".

## Authors' contributions

KKI carried out the assay design, sequence alignment and assay performance studies. SHQ, YM, DZ, MEM and JG-G assisted with the assay optimization and cloning. MS and YQ participated in acquiring the samples. MT participated in assay validation. KM, YQ, and YI participated in the conception and design of the studies. AK reviewed patient charts and confirmed clinical diagnoses. WTS reviewed histopathological findings to confirm clinical diagnoses. DDI reviewed test results and conducted further blind testing using this diagnosing system. All authors read and approved the final manuscript.

## Supplementary Material

Additional file 1 **Subtypes and GenBank accession numbers used in this study**. Accession numbers and subtype identity of the 271 BKV sequences used in this study are presented. (A) Subtype I accounted for 70% (190/271), (B) Subtype II accounted for <2% (4/271), (C) Subtype III accounted for <2% (4/271), and (D) Subtype IV accounted for 27% (73/271). Click here for file

Additional file 2 **Alignment of 271 BKV sequences corresponding to the VP2(VP3) C-terminus region**. Nucleotide alignment of 271 BKV sequences obtained from GenBank aligned to BKV Dunlop reference strain (V01108.1; nucleotides 1437 to 1679) is shown. The nucleotide range for each individual sequences are provided. Partial VP1 sequences were excluded from the alignment. The positions of the primers (underlined) and probe (boxed) are indicated on the BKV reference sequence. The subtype and subgroup identity for each sequence is provided. Subtypes were determined using the typing schema established by Jin et al. [31] and by Luo et al. [32]. The results were confirmed through phylogenetic analyses of the VP1 gene (data not shown). Subgroup identities for the 271 sequences were confirmed through phylogenetic analyses and trees constructed from whole genome sequences as previously described [31,33]. Phylogenetic trees were visualized using MEGA version 4 [34]. Click here for file
