# Supplementary material for: Development of a real-time quantitative PCR assay for detection of a stable genomic region of BK virus
Source: Virol J. 2010 Oct 29;7:295. doi: 10.1186/1743-422X-7-295 (PMC2989966; doi:10.1186/1743-422X-7-295)
Supplement: Additional file 1 — Subtypes and GenBank accession numbers used in this study. Accession numbers and subtype identity of the 271 BKV sequences used in this study are presented. (A) Subtype I accounted for 70% (190/271), (B) Subtype II accounted for <2% (4/271), (C) Subtype III accounted for <2% (4/271), and (D) Subtype IV accounted for 27% (73/271). [file 1743-422X-7-295-S1.DOC]

**A. Subtype I (n=190):** AB369092, AB365157, AB263938, AB263928, AB263926, AB263914, AB263913, AB263912, DQ989812, DQ989807, DQ989804, DQ989802, DQ305492, V01108, V01109, AB369094, AB369090, AB365177, AB365169, AB365163, AB301099, AB301095, AB301090, AB298947, AB263937, AB263934, AB263933, AB263932, AB263929, AB263927, AB263922, DQ989813, DQ989809, DQ989806, AB211373, AB211371, AB211374, AB211369, AY628238, AY628237, AY628225, AY628233, AY628232, AY628230, AY628229, AY628228, AY628227, AY628224, AY628231, AY628226, AB369091, AB369087, AB301103, AB301102, AB301100, AB301096, AB301094, AB301093, AB301091, AB301089, AB301088, AB301086, AB263936, AB263935, AB263924, AB263923, AB263921, AB263919, AB263918, AB263917, AB263915, DQ989810, DQ989805, DQ989803, DQ989801, DQ989799, DQ989797, DQ989796, AB260032, AB260031, AB260030, AB260029, AB260028, AB211370, AY628236, AY628235, AY628234, AB301092, AB301087, DQ989811, DQ989800, DQ989798, DQ989794, AB263925, DQ989808, AB369088, DQ989795, AB464958, AB365166, AB365156, AB365151, AB211377, AB211385, AB217920, AB464963, AB464962, AB464961, AB464960, AB464959, AB464957, AB464956, AB464955, AB464954, AB464953, AB485712, AB485711, AB485710, AB485709, AB485708, AB485707, AB485706, AB485705, AB485704, AB485703, AB485702, AB485701, AB485700, AB485699, AB485698, AB485697, AB485696, AB485695, AB485694, AB369101, AB369100, AB369099, AB369098, AB369097, AB369096, AB369095, AB365170, AB365176, AB365175, AB365174, AB365165, AB365164, AB365162, AB365161, AB365160, AB365159, AB365158, AB365155, AB365154, AB365153, AB365152, AB365148, AB365146, AB365145, AB365144, AB365137, AB365136, AB365135, AB365134, AB365133, AB365132, AB365131, AB301098, AB298946, AB298945, AB298944, AB298943, AB298942, AB298941, AB298940, AB263931, AB263930, AB211384, AB211383, AB211382, AB211381, AB211380, AB211378, AB211376, AB211375, AB211372, AB211379, AB217921, AB217918, AB217917, AB213487

**B. Subtype II (n=4):** AB301101, EF376992, AB263920, AB263916

**C. Subtype III (n=4):** AB365139, AB365130, AB211386, M23122

**D. Subtype IV (n=73):** AB365171, AB269869, AB269860, AB269859, AB269842, AB365149, AB269868, AB269862, AB269845, AB269841, AB269826, AB211389, AB365147, AB365143, AB365142, AB365140, AB269840, AB269837, AB269836, AB211390, AB211391, AB217919, AB365150, AB365141, AB365138, AB269851, AB269839, AB269838, AB269835, AB269834, AB211387, AB211388, AB365178, AB365173, AB365172, AB365168, AB365167, AB269867, AB269865, AB269864, AB269863, AB269861, AB269858, AB269856, AB269855, AB269854, AB269853, AB269852, AB269850, AB269846, AB269844, AB269843, AB269828, AB269827, AB369093, AB369089, AB301097, AB269866, AB269857, AB269849, AB269848, AB269847, AB269833, AB269832, AB269831, AB269830, AB269829, AB269825, AB269824, AB269823, AB269822, AB260034, AB260033
