# Supplementary material for: Development of a real-time quantitative PCR assay for detection of a stable genomic region of BK virus
Source: Virol J. 2010 Oct 29;7:295. doi: 10.1186/1743-422X-7-295 (PMC2989966; doi:10.1186/1743-422X-7-295)
Supplement: Additional file 2 — Alignment of 271 BKV sequences corresponding to the VP2(VP3) C-terminus region. Nucleotide alignment of 271 BKV sequences obtained from GenBank aligned to BKV Dunlop reference strain (V01108.1; nucleotides 1437 to 1679) is shown. The nucleotide range for each individual sequences are provided. Partial VP1 sequences were excluded from the alignment. The positions of the primers (underlined) and probe (boxed) are indicated on the BKV reference sequence. The subtype and subgroup identity for each sequence is provided. Subtypes were determined using the typing schema established by Jin et al. [31] and by Luo et al. [32]. The results were confirmed through phylogenetic analyses of the VP1 gene (data not shown). Subgroup identities for the 271 sequences were confirmed through phylogenetic analyses and trees constructed from whole genome sequences as previously described [31,33]. Phylogenetic trees were visualized using MEGA version 4 [34]. [file 1743-422X-7-295-S2.DOC]

**Sub- Sub-**

**Accession** **group type**

**V01108 1437** TCAGGAGAGTTTATAGAAAAAACTATTGCCCCAGGAGGTGCTAATCAAAGAACTGCTCCTCAATGGATGTTGCCTTTACTTCTAGGCCTGTACGGGACTGTAACACCTGCTCTTGAAGCATATGAAGATGGCCCCAACCAAAAGAAAAGGAGAGTGTCCAGGGGCAGCTCCCAAAAAGCCAAAGGAACCCGTGCAAGTGCCAAAACTACTAATAAAAGGAGGAGTAGAAGTTCTAGAAGTTAA **1679** Ia I

AB369092 1317 ................................................................................................................................................................................................................................................... 1559 Ia I

AB365157 1317 ................................................................................................................................................................................................................................................... 1559 Ia I

AB263938 1317 ................................................................................................................................................................................................................................................... 1559 Ia I

AB263928 1317 ................................................................................................................................................................................................................................................... 1559 Ia I

AB263926 1317 ................................................................................................................................................................................................................................................... 1559 Ia I

AB263914 1317 ................................................................................................................................................................................................................................................... 1559 Ia I

AB263913 1317 ................................................................................................................................................................................................................................................... 1559 Ia I

AB263912 1317 ................................................................................................................................................................................................................................................... 1559 Ia I

DQ989812 1050 ................................................................................................................................................................................................................................................... 1292 Ia I

DQ989807 1050 ................................................................................................................................................................................................................................................... 1292 Ia I

DQ989804 1050 ................................................................................................................................................................................................................................................... 1292 Ia I

DQ989802 1050 ................................................................................................................................................................................................................................................... 1292 Ia I

DQ305492 1474 ................................................................................................................................................................................................................................................... 1716 Ia I

AB369094 1317 ................................................................................................................................................................................................................................................... 1559 Ib-1 I

AB369090 1317 ................................................................................................................................................................................................................................................... 1559 Ib-1 I

AB365177 1317 ................................................................................................................................................................................................................................................... 1559 Ib-1 I

AB365169 1317 ................................................................................................................................................................................................................................................... 1559 Ib-1 I

AB365163 1317 ................................................................................................................................................................................................................................................... 1559 Ib-1 I

AB301099 1317 ................................................................................................................................................................................................................................................... 1559 Ib-1 I

AB301095 1317 ................................................................................................................................................................................................................................................... 1559 Ib-1 I

AB301090 1317 ................................................................................................................................................................................................................................................... 1559 Ib-1 I

AB298947 1317 ................................................................................................................................................................................................................................................... 1559 Ib-1 I

AB263937 1317 ................................................................................................................................................................................................................................................... 1559 Ib-1 I

AB263934 1317 ................................................................................................................................................................................................................................................... 1559 Ib-1 I

AB263933 1317 ................................................................................................................................................................................................................................................... 1559 Ib-1 I

AB263932 1317 ................................................................................................................................................................................................................................................... 1559 Ib-1 I

AB263929 1338 ................................................................................................................................................................................................................................................... 1580 Ib-1 I

AB263927 1317 ................................................................................................................................................................................................................................................... 1559 Ib-1 I

AB263922 1317 ................................................................................................................................................................................................................................................... 1559 Ib-1 I

DQ989813 1050 ................................................................................................................................................................................................................................................... 1292 Ib-1 I

DQ989809 1050 ................................................................................................................................................................................................................................................... 1292 Ib-1 I

DQ989806 1050 ................................................................................................................................................................................................................................................... 1292 Ib-1 I

AB211373 1317 ................................................................................................................................................................................................................................................... 1559 Ib-1 I

AB211371 1317 ................................................................................................................................................................................................................................................... 1559 Ib-1 I

AB211374 1317 ................................................................................................................................................................................................................................................... 1559 Ib-1 I

AB211369 1317 ................................................................................................................................................................................................................................................... 1559 Ib-1 I

AY628238 1416 ................................................................................................................................................................................................................................................... 1658 Ib-1 I

AY628237 1413 ................................................................................................................................................................................................................................................... 1655 Ib-1 I

AY628225 1425 ................................................................................................................................................................................................................................................... 1667 Ib-1 I

AY628233 1425 ................................................................................................................................................................................................................................................... 1667 Ib-1 I

AY628232 1425 ................................................................................................................................................................................................................................................... 1667 Ib-1 I

AY628230 1425 ................................................................................................................................................................................................................................................... 1667 Ib-1 I

AY628229 1425 ................................................................................................................................................................................................................................................... 1667 Ib-1 I

AY628228 1425 ................................................................................................................................................................................................................................................... 1667 Ib-1 I

AY628227 1425 ................................................................................................................................................................................................................................................... 1667 Ib-1 I

AY628224 1425 ................................................................................................................................................................................................................................................... 1667 Ib-1 I

AB464958 1317 ................................................................................................................................................................................................................................................... 1559 Ic I

AB365166 1317 ................................................................................................................................................................................................................................................... 1559 Ic I

AB365156 1317 ................................................................................................................................................................................................................................................... 1559 Ic I

AB365151 1317 ................................................................................................................................................................................................................................................... 1559 Ic I

AB211377 1309 ................................................................................................................................................................................................................................................... 1551 Ic I

AB211385 1317 ................................................................................................................................................................................................................................................... 1559 Ic I

AB217920 1317 ................................................................................................................................................................................................................................................... 1559 Ic I

AB464963 1317 ..........................................................................................................................................................................................C........................................................ 1559 Ic I

AB464962 1317 ..........................................................................................................................................................................................C........................................................ 1559 Ic I

AB464961 1317 ..........................................................................................................................................................................................C........................................................ 1559 Ic I

AB464960 1317 ..........................................................................................................................................................................................C........................................................ 1559 Ic I

AB464959 1317 ..........................................................................................................................................................................................C........................................................ 1559 Ic I

AB464957 1317 ..........................................................................................................................................................................................C........................................................ 1559 Ic I

AB464956 1317 ..........................................................................................................................................................................................C........................................................ 1559 Ic I

AB464955 1317 ..........................................................................................................................................................................................C........................................................ 1559 Ic I

AB464954 1317 ..........................................................................................................................................................................................C........................................................ 1559 Ic I

AB464953 1317 ..........................................................................................................................................................................................C........................................................ 1559 Ic I

AB485712 1460 ..........................................................................................................................................................................................C........................................................ 1702 Ic I

AB485711 1308 ..........................................................................................................................................................................................C........................................................ 1550 Ic I

AB485710 1460 ..........................................................................................................................................................................................C........................................................ 1702 Ic I

AB485709 1352 ..........................................................................................................................................................................................C........................................................ 1594 Ic I

AB485708 1350 ..........................................................................................................................................................................................C........................................................ 1592 Ic I

AB485707 1317 ..........................................................................................................................................................................................C........................................................ 1559 Ic I

AB485706 1350 ..........................................................................................................................................................................................C........................................................ 1592 Ic I

AB485705 1350 ..........................................................................................................................................................................................C........................................................ 1592 Ic I

AB485704 1350 ..........................................................................................................................................................................................C........................................................ 1592 Ic I

AB485703 1308 ..........................................................................................................................................................................................C........................................................ 1550 Ic I

AB485702 1460 ..........................................................................................................................................................................................C........................................................ 1702 Ic I

AB485701 1549 ..........................................................................................................................................................................................C........................................................ 1791 Ic I

AB485700 1536 ..........................................................................................................................................................................................C........................................................ 1778 Ic I

AB485699 1555 ..........................................................................................................................................................................................C........................................................ 1797 Ic I

AB485698 1554 ..........................................................................................................................................................................................C........................................................ 1796 Ic I

AB485697 1553 ..........................................................................................................................................................................................C........................................................ 1795 Ic I

AB485696 1317 ..........................................................................................................................................................................................C........................................................ 1559 Ic I

AB485695 1460 ..........................................................................................................................................................................................C........................................................ 1702 Ic I

AB485694 1317 ..........................................................................................................................................................................................C........................................................ 1559 Ic I

AB369101 1317 ..........................................................................................................................................................................................C........................................................ 1559 Ic I

AB369100 1317 ..........................................................................................................................................................................................C........................................................ 1559 Ic I

AB369099 1317 ..........................................................................................................................................................................................C........................................................ 1559 Ic I

AB369098 1317 ..........................................................................................................................................................................................C........................................................ 1559 Ic I

AB369097 1317 ..........................................................................................................................................................................................C........................................................ 1559 Ic I

AB369096 1317 ..........................................................................................................................................................................................C........................................................ 1559 Ic I

AB369095 1317 ..........................................................................................................................................................................................C........................................................ 1559 Ic I

AB365170 1317 ..........................................................................................................................................................................................C........................................................ 1559 Ic I

AB365176 1317 ..........................................................................................................................................................................................C........................................................ 1559 Ic I

AB365175 1317 ..........................................................................................................................................................................................C........................................................ 1559 Ic I

AB365174 1317 ..........................................................................................................................................................................................C........................................................ 1559 Ic I

AB365165 1317 ..........................................................................................................................................................................................C........................................................ 1559 Ic I

AB365164 1317 ..........................................................................................................................................................................................C........................................................ 1559 Ic I

AB365162 1317 ..........................................................................................................................................................................................C........................................................ 1559 Ic I

AB365161 1317 ..........................................................................................................................................................................................C........................................................ 1559 Ic I

AB365160 1317 ..........................................................................................................................................................................................C........................................................ 1559 Ic I

AB365159 1317 ..........................................................................................................................................................................................C........................................................ 1559 Ic I

AB365158 1317 ..........................................................................................................................................................................................C........................................................ 1559 Ic I

AB365155 1317 ..........................................................................................................................................................................................C........................................................ 1559 Ic I

AB365154 1317 ..........................................................................................................................................................................................C........................................................ 1559 Ic I

AB365153 1317 ..........................................................................................................................................................................................C........................................................ 1559 Ic I

AB365152 1317 ..........................................................................................................................................................................................C........................................................ 1559 Ic I

AB365148 1317 ..........................................................................................................................................................................................C........................................................ 1559 Ic I

AB365146 1317 ..........................................................................................................................................................................................C........................................................ 1559 Ic I

AB365145 1317 ..........................................................................................................................................................................................C........................................................ 1559 Ic I

AB365144 1317 ..........................................................................................................................................................................................C........................................................ 1559 Ic I

AB365137 1317 ..........................................................................................................................................................................................C........................................................ 1559 Ic I

AB365136 1317 ..........................................................................................................................................................................................C........................................................ 1559 Ic I

AB365135 1317 ..........................................................................................................................................................................................C........................................................ 1559 Ic I

AB365134 1331 ..........................................................................................................................................................................................C........................................................ 1573 Ic I

AB365133 1317 ..........................................................................................................................................................................................C........................................................ 1559 Ic I

AB365132 1317 ..........................................................................................................................................................................................C........................................................ 1559 Ic I

AB365131 1317 ..........................................................................................................................................................................................C........................................................ 1559 Ic I

AB301098 1317 ..........................................................................................................................................................................................C........................................................ 1559 Ic I

AB298946 1317 ..........................................................................................................................................................................................C........................................................ 1559 Ic I

AB298945 1317 ..........................................................................................................................................................................................C........................................................ 1559 Ic I

AB298944 1317 ..........................................................................................................................................................................................C........................................................ 1559 Ic I

AB298943 1317 ..........................................................................................................................................................................................C........................................................ 1559 Ic I

AB298942 1317 ..........................................................................................................................................................................................C........................................................ 1559 Ic I

AB298941 1317 ..........................................................................................................................................................................................C........................................................ 1559 Ic I

AB298940 1317 ..........................................................................................................................................................................................C........................................................ 1559 Ic I

AB263931 1317 ..........................................................................................................................................................................................C........................................................ 1559 Ic I

AB263930 1317 ..........................................................................................................................................................................................C........................................................ 1559 Ic I

AB211384 1317 ..........................................................................................................................................................................................C........................................................ 1559 Ic I

AB211383 1317 ..........................................................................................................................................................................................C........................................................ 1559 Ic I

AB211382 1317 ..........................................................................................................................................................................................C........................................................ 1559 Ic I

AB211381 1317 ..........................................................................................................................................................................................C........................................................ 1559 Ic I

AB211380 1317 ..........................................................................................................................................................................................C........................................................ 1559 Ic I

AB211378 1316 ..........................................................................................................................................................................................C........................................................ 1558 Ic I

AB211376 1317 ..........................................................................................................................................................................................C........................................................ 1559 Ic I

AB211375 1317 ..........................................................................................................................................................................................C........................................................ 1559 Ic I

AB211372 1317 ..........................................................................................................................................................................................C........................................................ 1559 Ic I

AB211379 1317 ..........................................................................................................................................................................................C........................................................ 1559 Ic I

AB217921 1317 ..........................................................................................................................................................................................C........................................................ 1559 Ic I

AB217918 1317 ..........................................................................................................................................................................................C........................................................ 1559 Ic I

AB217917 1317 ..........................................................................................................................................................................................C........................................................ 1559 Ic I

AB213487 1317 ..........................................................................................................................................................................................C........................................................ 1559 Ic I

AB365149 1308 .............................................................................G............................................................................................................C........................................................ 1550 IVa-2 IV

AB269868 1309 .............................................................................G............................................................................................................C........................................................ 1551 IVa-2 IV

AB269862 1289 .............................................................................G............................................................................................................C........................................................ 1531 IVa-2 IV

AB269845 1309 .............................................................................G............................................................................................................C........................................................ 1551 IVa-2 IV

AB269841 1309 .............................................................................G............................................................................................................C........................................................ 1551 IVa-2 IV

AB269826 1309 .............................................................................G............................................................................................................C........................................................ 1551 IVa-2 IV

AB211389 1308 .............................................................................G............................................................................................................C........................................................ 1550 IVa-2 IV

AB365171 1318 .............................................................................G..................................................................................................................................................................... 1560 IVa-1 IV

AB269869 1318 .............................................................................G..................................................................................................................................................................... 1560 IVa-1 IV

AB269842 1318 .............................................................................G..................................................................................................................................................................... 1560 IVa-1 IV

AB269860 1318 .............................................................................G..................................................................................................................................................................... 1560 IVa-1 IV

AB269859 1318 .............................................................................G..................................................................................................................................................................... 1560 IVa-1 IV

AB365147 1318 .............................................................................G..................................................................................................................................................................... 1560 IVb-1 IV

AB365143 1318 .............................................................................G..................................................................................................................................................................... 1560 IVb-1 IV

AB365142 1318 .............................................................................G..................................................................................................................................................................... 1560 IVb-1 IV

AB365140 1421 .............................................................................G..................................................................................................................................................................... 1663 IVb-1 IV

AB269840 1318 .............................................................................G..................................................................................................................................................................... 1560 IVb-1 IV

AB269837 1318 .............................................................................G..................................................................................................................................................................... 1560 IVb-1 IV

AB269836 1318 .............................................................................G..................................................................................................................................................................... 1560 IVb-1 IV

AB211390 1227 .............................................................................G..................................................................................................................................................................... 1469 IVb-1 IV

AB211391 1318 .............................................................................G..................................................................................................................................................................... 1560 IVb-1 IV

AB217919 1318 .............................................................................G..................................................................................................................................................................... 1560 IVb-1 IV

AB365150 1309 .............................................................................G..................................................................................................................................................................... 1551 IVb-2 IV

AB365141 1309 .............................................................................G..................................................................................................................................................................... 1551 IVb-2 IV

AB365138 1309 .............................................................................G..................................................................................................................................................................... 1551 IVb-2 IV

AB269851 1309 .............................................................................G..................................................................................................................................................................... 1551 IVb-2 IV

AB269839 1309 .............................................................................G..................................................................................................................................................................... 1551 IVb-2 IV

AB269838 1309 .............................................................................G..................................................................................................................................................................... 1551 IVb-2 IV

AB269835 1309 .............................................................................G..................................................................................................................................................................... 1551 IVb-2 IV

AB269834 1309 .............................................................................G..................................................................................................................................................................... 1551 IVb-2 IV

AB211387 1309 .............................................................................G..................................................................................................................................................................... 1551 IVb-2 IV

AB211388 1309 .............................................................................G..................................................................................................................................................................... 1551 IVb-2 IV

AB365178 1318 .............................................................................G..................................................................................................................................................................... 1560 IVc-1 IV

AB365173 1318 .............................................................................G..................................................................................................................................................................... 1560 IVc-1 IV

AB365172 1318 .............................................................................G..................................................................................................................................................................... 1560 IVc-1 IV

AB365168 1318 .............................................................................G..................................................................................................................................................................... 1560 IVc-1 IV

AB365167 1318 .............................................................................G..................................................................................................................................................................... 1560 IVc-1 IV

AB269867 1318 .............................................................................G..................................................................................................................................................................... 1560 IVc-1 IV

AB269865 1318 .............................................................................G..................................................................................................................................................................... 1560 IVc-1 IV

AB269864 1318 .............................................................................G..................................................................................................................................................................... 1560 IVc-1 IV

AB269863 1318 .............................................................................G..................................................................................................................................................................... 1560 IVc-1 IV

AB269861 1318 .............................................................................G..................................................................................................................................................................... 1560 IVc-1 IV

AB269858 1318 .............................................................................G..................................................................................................................................................................... 1560 IVc-1 IV

AB269856 1318 .............................................................................G..................................................................................................................................................................... 1560 IVc-1 IV

AB269855 1318 .............................................................................G..................................................................................................................................................................... 1560 IVc-1 IV

AB269854 1318 .............................................................................G..................................................................................................................................................................... 1560 IVc-1 IV

AB269853 1318 .............................................................................G..................................................................................................................................................................... 1560 IVc-1 IV

AB269852 1318 .............................................................................G..................................................................................................................................................................... 1560 IVc-1 IV

AB269850 1318 .............................................................................G..................................................................................................................................................................... 1560 IVc-1 IV

AB269846 1317 .............................................................................G..................................................................................................................................................................... 1559 IVc-1 IV

AB269844 1309 .............................................................................G..................................................................................................................................................................... 1551 IVc-1 IV

AB269843 1318 .............................................................................G..................................................................................................................................................................... 1560 IVc-1 IV

AB269828 1318 .............................................................................G..................................................................................................................................................................... 1560 IVc-1 IV

AB269827 1318 .............................................................................G..................................................................................................................................................................... 1560 IVc-1 IV

AB369093 1318 .............................................................................G..................................................................................................................................................................... 1560 IVc-2 IV

AB369089 1318 .............................................................................G..................................................................................................................................................................... 1560 IVc-2 IV

AB301097 1318 .............................................................................G..................................................................................................................................................................... 1560 IVc-2 IV

AB269866 1318 .............................................................................G..................................................................................................................................................................... 1560 IVc-2 IV

AB269857 1318 .............................................................................G..................................................................................................................................................................... 1560 IVc-2 IV

AB269849 1318 .............................................................................G..................................................................................................................................................................... 1560 IVc-2 IV

AB269848 1318 .............................................................................G..................................................................................................................................................................... 1560 IVc-2 IV

AB269847 1318 .............................................................................G..................................................................................................................................................................... 1560 IVc-2 IV

AB269833 1318 .............................................................................G..................................................................................................................................................................... 1560 IVc-2 IV

AB269832 1318 .............................................................................G..................................................................................................................................................................... 1560 IVc-2 IV

AB269831 1318 .............................................................................G..................................................................................................................................................................... 1560 IVc-2 IV

AB269830 1318 .............................................................................G..................................................................................................................................................................... 1560 IVc-2 IV

AB269829 1318 .............................................................................G..................................................................................................................................................................... 1560 IVc-2 IV

AB269825 1318 .............................................................................G..................................................................................................................................................................... 1560 IVc-2 IV

AB269824 1318 .............................................................................G..................................................................................................................................................................... 1560 IVc-2 IV

AB269823 1318 .............................................................................G..................................................................................................................................................................... 1560 IVc-2 IV

AB269822 1318 .............................................................................G..................................................................................................................................................................... 1560 IVc-2 IV

AB260034 1318 .............................................................................G..................................................................................................................................................................... 1560 IVc-2 IV

AB260033 1318 .............................................................................G..................................................................................................................................................................... 1560 IVc-2 IV

AB301101 1307 .............................................................................G..................................................................................................................................................................... 1549 II

EF376992 1307 .............................................................................G..................................................................................................................................................................... 1549 II

AB263920 1307 .............................................................................G..................................................................................................................................................................... 1549 II

AB263916 1316 .............................................................................G..................................................................................................................................................................... 1558 II

AB365139 1316 .............................................................................G..................................................................................................................................................................... 1558 III

AB365130 1316 .............................................................................G..................................................................................................................................................................... 1558 III

AB211386 1316 .............................................................................G..................................................................................................................................................................... 1558 III

M23122 1283 .............................................................................G..................................................................................................................................................................... 1525 III

AB369091 1317 ..........................................................................................................................................A........................................................................................................ 1559 Ib-2 I

AB369087 1317 ..........................................................................................................................................A........................................................................................................ 1559 Ib-2 I

AB301103 1317 ..........................................................................................................................................A........................................................................................................ 1559 Ib-2 I

AB301102 1317 ..........................................................................................................................................A........................................................................................................ 1559 Ib-2 I

AB301100 1317 ..........................................................................................................................................A........................................................................................................ 1559 Ib-2 I

AB301096 1317 ..........................................................................................................................................A........................................................................................................ 1559 Ib-2 I

AB301094 1317 ..........................................................................................................................................A........................................................................................................ 1559 Ib-2 I

AB301093 1317 ..........................................................................................................................................A........................................................................................................ 1559 Ib-2 I

AB301091 1317 ..........................................................................................................................................A........................................................................................................ 1559 Ib-2 I

AB301089 1317 ..........................................................................................................................................A........................................................................................................ 1559 Ib-2 I

AB301088 1317 ..........................................................................................................................................A........................................................................................................ 1559 Ib-2 I

AB301086 1317 ..........................................................................................................................................A........................................................................................................ 1559 Ib-2 I

AB263936 1317 ..........................................................................................................................................A........................................................................................................ 1559 Ib-2 I

AB263935 1317 ..........................................................................................................................................A........................................................................................................ 1559 Ib-2 I

AB263924 1317 ..........................................................................................................................................A........................................................................................................ 1559 Ib-2 I

AB263923 1317 ..........................................................................................................................................A........................................................................................................ 1559 Ib-2 I

AB263921 1317 ..........................................................................................................................................A........................................................................................................ 1559 Ib-2 I

AB263919 1317 ..........................................................................................................................................A........................................................................................................ 1559 Ib-2 I

AB263918 1317 ..........................................................................................................................................A........................................................................................................ 1559 Ib-2 I

AB263917 1317 ..........................................................................................................................................A........................................................................................................ 1559 Ib-2 I

AB263915 1317 ..........................................................................................................................................A........................................................................................................ 1559 Ib-2 I

DQ989810 1050 ..........................................................................................................................................A........................................................................................................ 1292 Ib-2 I

DQ989805 1050 ..........................................................................................................................................A........................................................................................................ 1292 Ib-2 I

DQ989803 1050 ..........................................................................................................................................A........................................................................................................ 1292 Ib-2 I

DQ989801 1050 ..........................................................................................................................................A........................................................................................................ 1292 Ib-2 I

DQ989799 1050 ..........................................................................................................................................A........................................................................................................ 1292 Ib-2 I

DQ989797 1050 ..........................................................................................................................................A........................................................................................................ 1292 Ib-2 I

DQ989796 1050 ..........................................................................................................................................A........................................................................................................ 1292 Ib-2 I

AB260032 1317 ..........................................................................................................................................A........................................................................................................ 1559 Ib-2 I

AB260031 1317 ..........................................................................................................................................A........................................................................................................ 1559 Ib-2 I

AB260030 1317 ..........................................................................................................................................A........................................................................................................ 1559 Ib-2 I

AB260029 1317 ..........................................................................................................................................A........................................................................................................ 1559 Ib-2 I

AB260028 1317 ..........................................................................................................................................A........................................................................................................ 1559 Ib-2 I

AB211370 1278 ..........................................................................................................................................A........................................................................................................ 1520 Ib-2 I

AY628236 1425 ..........................................................................................................................................A........................................................................................................ 1667 Ib-2 I

AY628235 1425 ..........................................................................................................................................A........................................................................................................ 1667 Ib-2 I

AY628234 1376 ..........................................................................................................................................A........................................................................................................ 1618 Ib-2 I

AB301092 1317 ..........................................................................................................................................A....................................................................G................................... 1559 Ib-2 I

AB301087 1317 ..........................................................................................................................................A....................................................................G................................... 1559 Ib-2 I

DQ989811 1050 ..........................................................................................................................................A....................................................................G................................... 1292 Ib-2 I

DQ989800 1050 ..........................................................................................................................................A....................................................................G................................... 1292 Ib-2 I

DQ989798 1050 ..........................................................................................................................................A....................................................................G................................... 1292 Ib-2 I

DQ989794 1050 ..........................................................................................................................................A....................................................................G................................... 1292 Ib-2 I

AB263925 1308 ..........................................................................................................................................A.................................................................G...................................... 1550 Ib-2 I

DQ989808 1050 ..........................................................................................................................................A....A................................................................................................... 1292 Ib-2 I

AB369088 1317 ........A.................................................................................................................................A........................................................................................................ 1559 Ib-2 I

DQ989795 1050 ..........................................................................................................................................A...................................................A.................................................... 1292 Ib-2 I

V01109 4559 .........................................................................................................................G......................................................................................................................... 4801 Ia I

AY628231 1425 .....................................................................................................................................................................................G............................................................. 1667 Ib-1 I

AY628226 1425 ........................................................................................................................................G.......................................................................................................... 1667 Ib-1 I
